# Hepatic stem cells with self-renewal and liver repopulation potential are harbored in CDCP1-positive subpopulations of human fetal liver cells

**DOI:** 10.1186/s13287-017-0747-3

**Published:** 2018-02-05

**Authors:** Ran-Ran Zhang, Yun-Wen Zheng, Bin Li, Yun-Zhong Nie, Yasuharu Ueno, Tomonori Tsuchida, Hideki Taniguchi

**Affiliations:** 1 Department of Regenerative Medicine, Graduate School of Medicine, Yokohama City University, 3-9 Fuku-ura, Kanazawa-ku, Yokohama, Kanagawa 236-0004 Japan; 2 Department of Advanced Gastroenterological Surgical Science and Technology, Faculty of Medicine, University of Tsukuba, Tsukuba, 305-8575 Japan; 3 Research Center of Stem Cells and Regenerative Medicine, Jiangsu University Hospital, Zhenjiang, Jiangsu 212001 China; 4 Oregon Stem Cell Center, Oregon Health and Science University, Portland, OR 97239 USA; 5 Advanced Medical Research Center, Yokohama City University, 3-9 Fuku-ura, Kanazawa-ku, Yokohama, Kanagawa 236-0004 Japan; 6 Department of Gastroenterology, Hepatology & Nutrition, Developmental Biology and Center for Stem Cell and Organoid Medicine (CuSTOM), Cincinnati Children’s Hospital Medical Center, 3333 Burnet Avenue, Cincinnati, OH 45229 USA

**Keywords:** Hepatic stem cells, Transplantation, Drug metabolism

## Abstract

**Background:**

Mature human hepatocytes are critical in preclinical research and therapy for liver disease, but are difficult to manipulate and expand in vitro. Hepatic stem cells (HpSCs) may be an alternative source of functional hepatocytes for cell therapy and disease modeling. Since these cells play an import role in regenerative medicine, the precise characterization that determines specific markers used to isolate these cells as well as whether they contribute to liver regeneration still remain to be shown.

**Method:**

In this study, human HpSCs were isolated from human primary fetal liver cells (FLCs) by flow cytometry using CDCP1, CD90, and CD66 antibodies. The isolated CDCP1^+^CD90^+^CD66^–^ HpSCs were cultured on dishes coated with type IV collagen in DMEM nutrient mixture F-12 Ham supplemented with FBS, human γ-insulin, nicotinamide, dexamethasone, and l-glutamine for at least 2 weeks, and were characterized by transcriptomic profiling, quantitative real-time PCR, immunocytochemistry, and in-vivo transplantation.

**Results:**

The purified CDCP1^+^CD90^+^CD66^–^ subpopulation exhibited clonal expansion and self-renewal capability, and bipotential capacity was further identified in single cell-derived colonies containing distinct hepatocytes and cholangiocytes. Moreover, in-vivo liver repopulation assays demonstrated that human CDCP1^+^CD90^+^CD66^–^ HpSCs repopulated over 90% of the mouse liver and differentiated into functional hepatocytes with drug metabolism activity.

**Conclusions:**

We identified a human hepatic stem/progenitor population in the CDCP1^+^CD90^+^CD66^–^ subpopulation in human FLCs, indicating CDCP1 marker could potentially be utilized to identify and isolate HpSCs for further cytotherapy of liver disease.

**Electronic supplementary material:**

The online version of this article (doi:10.1186/s13287-017-0747-3) contains supplementary material, which is available to authorized users.

## Background

Liver organ transplants are the only available and efficient treatment for patients with end-stage liver failure; however, there is an increasing shortage of liver donors [1, 2]. Hepatocyte transplantation has become a viable alternative rescue [3, 4]. However, adult hepatocytes show minimal proliferative activity in vivo, which is consistent with the absence of hepatocellular proliferation in the normal liver, and are difficult to cryopreserve [5, 6]. Therefore, human adult hepatocytes have limited applications in physiopathology [7, 8], pharmacology [9], and toxicology [10], and the development of new alternative sources of human hepatocytes is essential. Hepatocyte-like cells (iHeps) can be derived from fibroblasts by direct reprogramming [11, 12] or from human induced pluripotent stem cells (iPSCs) [13] by differentiation; however, incomplete conversion causes impaired hepatocyte function [14] and incomplete iPSC reprogramming leads to carcinogenesis in vivo, thereby limiting clinical applications of these cell types [15]. Human hepatic stem cells (HpSCs) from the liver lineage have therefore become an attractive alternative cell source to primary hepatocytes.

HpSCs separated from fetal and postnatal livers could give rise to differentiated hepatocytes in vitro and more mature hepatocytes in vivo, providing an opportunity to overcome the obstacles already described [16, 17]. Indeed, many researchers have isolated, purified, and characterized HpSCs from human and rodent livers using flow cytometry. HpSCs have been shown to express CD29, CD49f [18], CD13 [19], CD117, CD133, EpCAM [20], and DLK [21], as well as markers of both hepatocyte and cholangiocyte lineages (such as albumin (ALB), alpha-fetoprotein (AFP), cytokeratin 18 (CK18), and CK19 [22]) and, more importantly, markers of both hematopoietic and mesenchymal stem cells (i.e., CD34, CD45, CD90, and CD109) [16, 23]. Notably, HpSCs that express a broad spectrum of markers have been identified previously; however, the specific marker expression profile that can be used to identify and isolate HpSCs from human primary fetal liver cells (FLCs) is still unclear.

CD90 (Thy-1), which was first reported in hematopoietic [24] and mesenchymal [25] stem cells, has also been identified as an oval cell marker [26] and is expressed in hepatic cancer stem cells of human liver cancer [27] as well as in hepatic stem/progenitor cells during liver development, but is rarely detected in adult liver cells [22, 28]. However, only CD90 is insufficiently sensitive or specific to be a marker for human HpSC isolation.

CUB domain-containing protein 1 (CDCP1) may be involved in cell adhesion or extracellular matrix interaction, and the expression of CDCP1 has been shown to correlate with the metastasis of carcinoma cells, such as in cancers of the colon [29], lung [30], breast [31], and pancreas [32]. In addition, numerous studies have shown that CDCP1 could also phenotypically identify hematopoietic stem cells [33–35] and mesenchymal and neural stem cells [34]. However, no studies have investigated the role of CDCP1 on HpSC isolation from human primary FLCs.

In the present study, we sought to isolate and characterize human HpSCs to determine their capacity for self-renewal and liver repopulation. Our data support that CDCP1 is a novel critical HpSC marker in combination with CD90 and CD66 expression.

## Results

### CDCP1 is a candidate marker for isolating clonal HpSCs from human primary FLCs

First, we confirmed whether human primary FLCs exhibited HpSC features. Under the clonal colony-forming assay, human primary FLCs grew in cell colonies with densities as low as 100 cells/cm^2^ on collagen IV-coated plates (Additional file 1: Figure S1A). Furthermore, cells coexpressing ALB and CK19 or AFP and CK19 were found in human primary FLCs, demonstrating the potential to give rise to hepatocytes and cholangiocytes (Additional file 1: Figure S1B). Our previous finding showed that mouse HpSCs were enriched in CD29^high^CD49f^+/low^ mouse primary FLCs (c-Kit^–^CD45^–^Ter119^–^ cells) [36], and in human primary FLCs the CD29^high^CD49f^+/low^ fraction exhibited a profile similar to that in mouse primary FLCs (Additional file 1: Figure S1C), further confirming that HpSCs might exist in human primary FLCs. To identify specific cell surface markers correlated with HpSC isolation, we utilized antigenic expression profiling by flow cytometry of hepatic and/or stem cell-related markers reported previously. These data revealed that human primary FLCs were highly enriched in CD29 (98.7%), CD49f (89.8%), CD24 (84.3%), and CD44 (99.8%), but negative for CD54, CD117, CD138, CD133, and CD140a. The oval cell marker CD90 was also expressed in about 40.1% of human primary FLCs. Additionally, we identified another candidate marker, CDCP1, which was expressed in 18.5% of human primary FLCs, suggesting that CDCP1 may be a novel and specific marker of HpSC isolation (Additional file 1: Figure S1D).

**Fig. 1:**
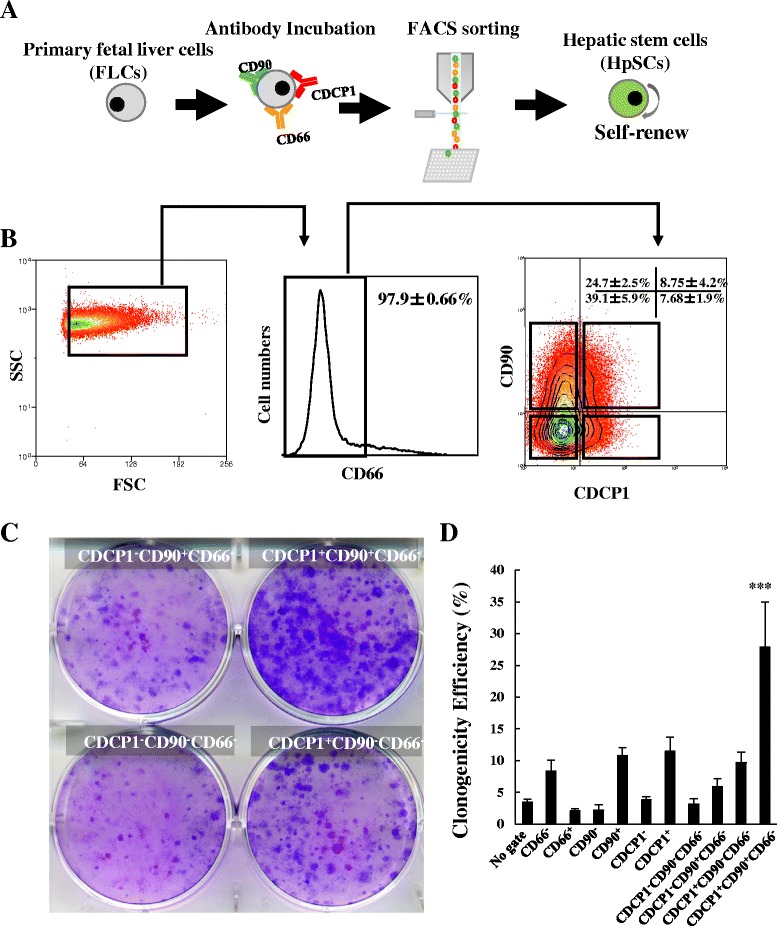
A putative human HpSC population was identified in the CDCP1^+^CD90^+^CD66^–^ subpopulation. **a** Schematic of the HpSC derivation protocol. **b** Human primary FLCs costained with human antibodies against CD66, CDCP1, and CD90 for flow cytometry analysis. CD66^–^ human primary FLCs were reanalyzed into four fractions based on the expression of CDCP1 and CD90: CDCP1^–^CD90^+^CD66^–^, CDCP1^+^CD90^+^CD66^–^, CDCP1^–^CD90^–^CD66^–^, and CDCP1^+^CD90^–^CD66^–^. Numbers represent the percentage of each fraction in CD66^–^ human primary FLCs (*n* = 7 independent experiments). **c** Four fractionated cell populations were sorted onto collagen IV-coated plates at a density of 100 cells/cm^2^. After 18 days of culture, colony-forming capabilities of the cell populations were characterized by Giemsa staining. **d** Efficiency of large-colony formation (containing more than 100 cells per colony) derived from clonal cell sorting as one cell per well of a 96-well plate determined from each fractionated subpopulation on day 18. Results presented as mean colony count ± SD (*n* = 4 independent experiments). See also Additional file 1: Figure S1, Additional file 2: Figure S2, and Additional file 3: Figure S3. FACS fluorescence-activated cell sorting, CDCP1 CUB domain-containing protein 1, SSC side scatter, FSC forward scatter. ***P < 0.0001

Previous research has shown that CD90 is expressed in hepatic stem/progenitor cells from adult or fetal livers [28]. Therefore, to confirm our hypothesis that CDCP1 is a specific marker of HpSC isolation, we fractionated human primary FLCs by flow cytometry using CDCP1 [34], combined with CD90 and CD66 (Fig. 1a), yielding four subpopulations of CDCP1^–^CD90^+^CD66^–^ (24.7 ± 2.5%), CDCP1^+^CD90^+^CD66^–^ (8.75 ± 4.2%), CDCP1^–^CD90^–^CD66^–^ (39.1 ± 5.9%), and CDCP1^+^CD90^–^CD66^–^ (7.68 ± 1.9%) (Fig. 1b); here we use CD66 to remove differentiated cells [37]. Low-density cell culture revealed that the CDCP1^+^CD90^+^CD66^–^ subpopulation yielded the most colonies as compared with other three subpopulations (Fig. 1c). Moreover, a clonal cell sorting assay (one cell per well in a 96-well plate) was performed to quantify the clonogenicity efficiency according to 11 combinations of CD66, CD90, and CDCP1. The efficiency of large colony formation was higher in CDCP1-positive fractions, including CDCP1^+^CD90^+^CD66^–^ (27.9 ± 8.6%, about 9-fold higher than the ungated subpopulation) and CDCP1^+^CD90^–^CD66^–^ (9.7 ± 2.0%) subpopulations, than in CDCP1-negative fractions, including CDCP1^–^CD90^+^CD66^–^ (5.9 ± 1.6%) and CDCP1^–^CD90^–^CD66^–^ (3.1 ± 1.0%) subpopulations (Fig. 1d). We continually analyzed antigenic profiling of HpSC markers on CDCP1^+^CD90^+^CD66^–^ subpopulations and found that CD13, a specific HpSC marker, was expressed in more than 80% of the CDCP1^+^CD90^+^CD66^–^ subpopulations. Moreover, more than 90% of the CDCP1^+^CD90^+^CD66^–^ subpopulations were positive for CD24, CD49f, CD44, CD55, and CD166, and negative for CD117, LGR5, CD45, and CD56 (Additional file 2: Figure S2). In contrast, other stem cell markers showed relatively lower positive rates: CD34 (2.4%), CD54 (0.1%), CD138 (3.1%), CD140a (2.7%), EpCAM (6.1%), and DLK (0.6%). Moreover, hepatitis C virus (HCV) receptors CD81 [38] and LDLR, were also positive in CDCP1^+^CD90^+^CD66^–^ subpopulations (99.5% and 26.1%, respectively; Additional file 2: Figure S2), indicating that the CDCP1^+^CD90^+^CD66^–^ subpopulation belongs to the hepatocyte lineage and may have relevance to the field of virology. From qPCR (Fig. 3a) and microarray data (Additional file 3: Figure S3A), we noted that the target genes of the Wnt/β-catenin signal pathway such as *CAV1*, *PTEN*, *CTNNB1*, *MYC*, *RAC1*, *BCL9L*, *GSK3A*, *FZD6*, *WNT5A*, *RTF1*, *SNAI2*, *CD44*, and *DKK1* were enriched in CDCP1^+^CD90^+^CD66^–^ HpSCs, which is consistent with other studies in which the Wnt/β-catenin pathway was shown to drive the HpSC population [39] and liver development/regeneration [40, 41]. When we detected cell surface marker genes (Additional file 3: Figure S3B) and stem cell-related genes (Additional file 3: Figure S3C) with the microarray, we found enhanced expression of some genes, including *HAS2*, *KDR*, *PTGER2*, *GNB1L*, *FAP*, *IL6*, *SERPINE2*, *MET*, *OSMR*, *CDH2*, *CD44*, *GATA2*, and *GATA3*. These results could provide valuable markers for comprehensively tracing and understanding HpSCs in the developing liver.

**Fig. 2:**
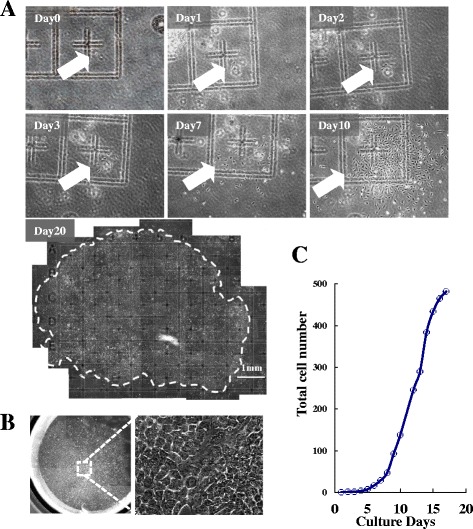
Clonal expansion of an isolated single CDCP1^+^CD90^+^ CD66^–^ HpSC. **a** Time tracing of a single CDCP1^+^CD90^+^CD66^–^ HpSC-derived colony. White arrow indicates single cell proliferation traced from day 0 to day 10, and the colony on day 20 is indicated by a white dashed line. **b** Representative image of clonal colony size and morphology from a single CDCP1^+^CD90^+^CD66^–^ HpSC in a 96-well plate. **c** Number of cells within a single HpSC-derived colony counted directly. Data shown as a clonal proliferation curve

To confirm the clonogenicity from a single putative stem cell, cell-tracing analysis was performed. Interestingly, large colonies contained more than 40,000 cells at day 20 (Fig. 2a) and showed a typical hepatocyte morphology (Fig. 2b) and strong single cell expansion potential (Fig. 2c), indicating that CDCP1 played an important role in colony formation and cell expansion. The cell proliferation tracing demonstrated that the doubling time of the cultured CDCP1^+^CD90^+^CD66^–^ subpopulation was 27.6 ± 4.7 h, consistent with a previous report [42]. These data suggest that clonal and proliferative HpSCs were isolated as the CDCP1^+^CD90^+^CD66^–^ subpopulation from human primary FLCs.

### CDCP1^+^CD90^+^CD66^–^ HpSCs had bipotential and self-renewal capabilities

**Fig. 3:**
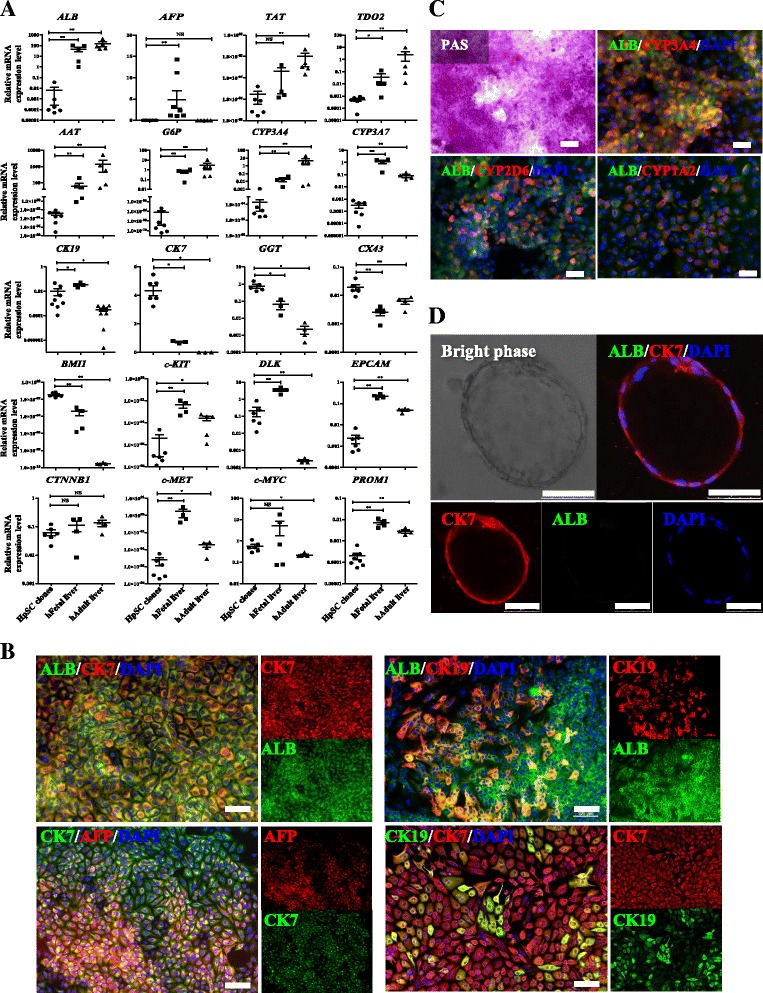
Bipotential differentiation capabilities of single HpSC-derived clones. **a** qPCR analysis of hepatocyte markers, cholangiocyte markers, and stem cell-related markers. HpSC clones, FACS-sorted single HpSC-derived clones after culture for 14 days; hFetal liver, samples from human primary FLCs; hAdult liver, samples from human adult liver cells. Results shown as mean ± SD (*n* = 3 independent experiments). Mann–Whitney test, *P < 0.05, **P < 0.001, NS no significant difference. **b** Immunocytochemical staining of hepatocyte markers of ALB and AFP and cholangiocyte-specific markers CK7 and CK19 in single human HpSC-derived colonies after culture for 40 days. Nuclei counterstained with DAPI. Scale bars: 100 μm. **c** Hepatocyte differentiation capability of single HpSC-derived colonies characterized after long-term culture for up to day 90 in vitro by Periodic acid–Schiff (PAS) staining and by positive immunocytochemical staining with human ALB, human CYP3A4, human CYP2D6, and human CYP1A2. Nuclei counterstained with DAPI. Scale bars: 100 μm. **d** Cholangiocytic cyst formation of single HpSC-derived colonies. FACS-sorted single HpSC-derived colonies were replated in an extracellular matrix gel, and several epithelial cysts were formed after culture for 14 days. CK7 was positively detected in cysts. Cells stained with antibodies against ALB and CK7. Nuclei counterstained with DAPI. Scale bars: 50 μm. Experiments were performed at least three times, representative data are shown. HpSC hepatic stem cell, ALB Albumin, CK cytokeratin

To investigate whether CDCP1^+^CD90^+^CD66^–^ HpSCs possessed hepatic bipotential capability and cholangiocytic differentiation ability, we evaluated single HpSC-derived clones (one clone per well in a 96-well plate). On culture day 14, single HpSC-derived clones were selected for gene expression analysis (Fig. 3a). qPCR analysis showed that all hepatic differentiation markers except for *AFP*, *ALB*, and *TAT* were detected, in addition to *TDO2*, *AAT*, *G6P*, *CYP3A4*, and *CYP3A7*. The cholangiocytic markers, including *CK19*, *CK7*, *GGT*, and *CX43*, were also detected, along with the stem cell-related markers (*BMI1*, *c*-*KIT*, *DLK*, *EPCAM*, *CTNNB1*, *c*-*MET*, *c*-*MYC*, and *PROM1*). These results indicated that each CDCP1^+^CD90^+^CD66^–^ HpSC has hepatic stem potential and could be differentiated into hepatic and cholangiocytic lineage cells in vitro. Additionally, after in-vitro culture of single CDCP1^+^CD90^+^CD66^–^ HpSC-derived clones for up to 40 days, immunostaining results showed that ALB-positive cells were colocalized with CK7 and CK19. Nearly all AFP-positive cells coexpressed CK7. A minor subpopulation of cells expressed both CK7 and CK19, representing more differentiated cholangiocytes (Fig. 3b). These data indicate that single HpSCs generated cell types including more mature hepatocytes and cholangiocytes in clonal culture. The generated hepatocytes displayed functional characteristics of mature hepatocytes, including glycogen storage and cytochrome P450 protein expression (Fig. 3c). Collectively, these results clearly showed that CDCP1^+^CD90^+^CD66^–^ HpSCs could differentiate into hepatocytes.

When single CDCP1^+^CD90^+^CD66^–^ HpSC-derived colonies were replated in a three-dimensional (3D) gel culture system containing 1.2 mg/ml collagen I and 40% Matrigel [43, 44], after 14 days of culture, many epithelial cysts were formed. Some of the epithelial cysts expressed CK7 but did not express the hepatic marker ALB (Fig. 3d). These data suggest that our isolated HpSCs were bipotential and could give rise to hepatocytes and cholangiocytes in vitro under suitable conditions.

**Fig. 4:**
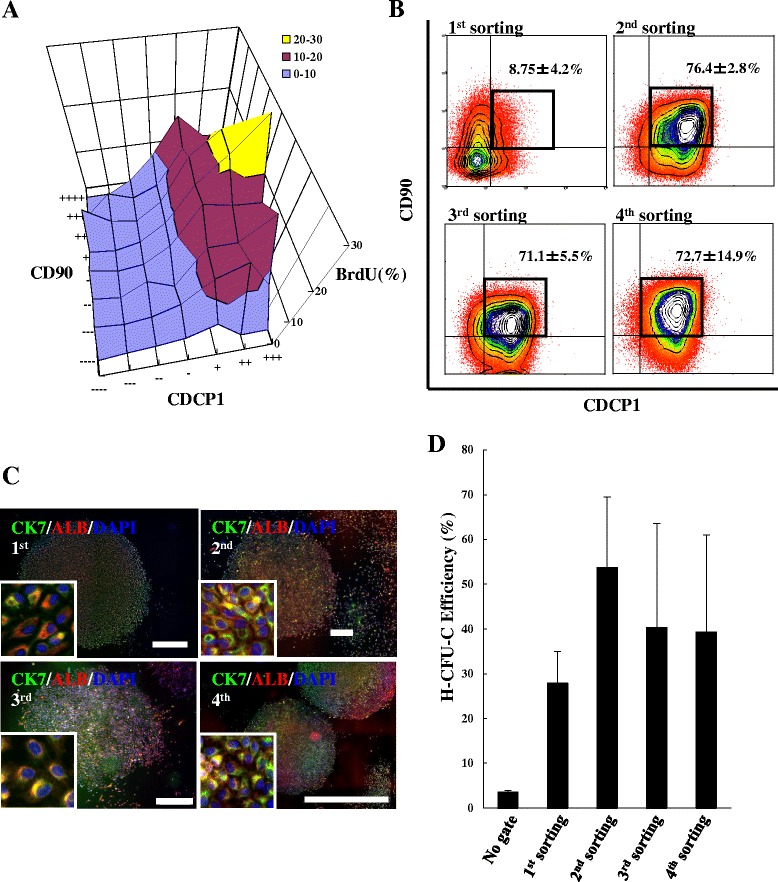
Self-renewal potential of HpSCs during serial sorting. **a** Proliferation of human HpSCs detected by BrdU labeling. *X*, *Y*, and *Z* axes indicate percentages of CDCP1-positive, CD90-positive, and BrdU-positive cells, respectively. **b** Characteristics of CDCP1^+^CD90^+^ fractions after serial sorting by flow cytometry. Primary cells from the first sorting, human FLCs; primary cells from the second, third, and fourth resortings, first, second, third sorting-derived human HpSCs. Numbers represent mean percentages of CDCP1^+^CD90^+^ cells ± SD (*n* = 4 independent experiments). **c** Clones derived from the first, second, third, and fourth sorted single HpSCs were positively stained with human ALB (red) and human CK7 (green). White-boxed image shows a high-magnification image. Scale bars: 500 μm. **d** Hepatic colony forming units in culture (H-CFU-C) efficiency analyzed in human HpSCs from the first, second, third, and fourth sorting. Results shown as mean percentages of colony counts ± SD (*n* = 3 independent experiments). ALB Albumin, BrdU 5-bromo-2-deoxyuridine, CK cytokeratin

5-Bromo-2-deoxyuridine (BrdU) was used to label human primary FLCs and to detect dividing cells by flow cytometry. The CDCP1^+^CD90^+^CD66^–^ HpSCs exhibited higher BrdU-positive rates (27.3%) than any other subpopulation (Fig. 4a), suggesting enrichment of highly proliferative and self-renewal cells. Next, we used serial sorting and single cell clonal assays to investigate the self-renewal potential of HpSCs in vitro. The first single cell sorting-derived HpSC colonies were replated into new culture plates. After 14 days of culture, the subcultured clones gradually expanded to reach confluence and were subsequently subjected to single cell sorting and culture again. After sorting four times, expanded HpSCs were enriched, and FACS patterns resembled the parental cell subpopulation from the second to fourth sorting (Fig. 4b). The CFU-C efficiency was relatively stable (Fig. 4d). Furthermore, single HpSC-derived colonies from the first to fourth serial sorting were large and round, with dual ALB and CK7-positive cells (Fig. 4c). More than 70% of cells in serial single cell sorting-derived HpSC colonies coexpressed ALB and CK7 (Table 1). The characteristics described indicated that CDCP1^+^CD90^+^CD66^–^ HpSCs have self-renewal ability with maintained H-CFU-C efficiency and ALB^+^CK7^+^ bipotential capability during serial sorting and subculture.

**Table 1:** Bipotential capability in single HpSC-derived colonies during serial sorting

Serial sorting	Single ALB^+^ (%)	Single CK7^+^ (%)	Double negative (%)	Double positive (%)
First	0.3 ± 0.9	18.6 ± 13.0	9.1 ± 4.7	72.0 ± 11.8
Second	0.1 ± 0.1	10.3 ± 6.6	8.1 ± 5.3	81.5 ± 9.8
Third	03 ± 0.6	2.7 ± 3.0	6.7 ± 4.0	90.3 ± 2.5
Fourth	0.0	12.9 ± 9.1	9.6 ± 7.4	77.5 ± 10.8

Immunostaining of human ALB and human CK7 in HpSC colonies derived from the first, second, third, and fourth sorting performed as described in Fig. 4c. Numbers represent percentage of ALB and/or CK7 stained or not stained in each colony. Results shown as mean percentage ± SD (*n* = 3 independent experiments)

*ALB* Albumin, *CK* cytokeratin, *HpSC* hepatic stem cell

**Fig. 5:**
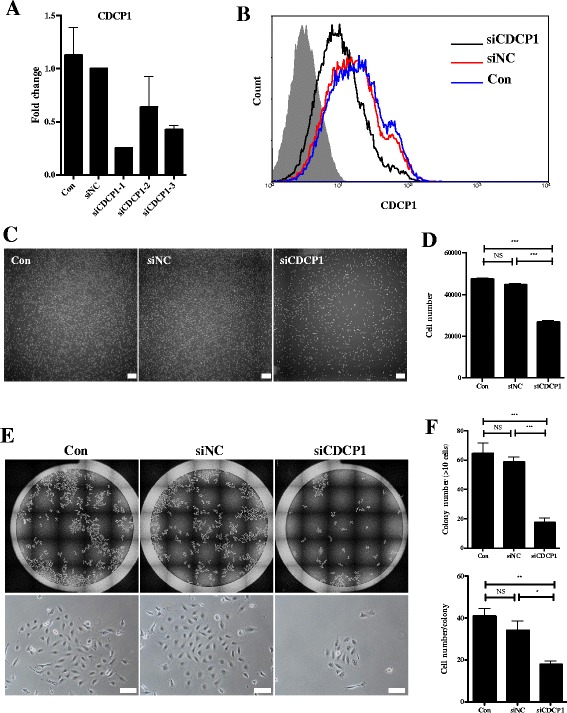
CDCP1 knockout inhibits cell proliferation and colony-forming capabilities in HpSCs. **a** qPCR analysis of the *CDCP1* mRNA expression level in siRNA-treated HpSCs. Con, untransfected HpSCs; siNC, HpSCs transfected with negative control siRNA; siCDCP1-1, siCDCP1-2, and siCDCP1-3, HpSCs transfected with siCDCP1. Total RNA was isolated 48 h after siRNA transfection. Results shown as mean fold change ± SD (*n* = 3 independent experiments). **b** FACS analysis of the CDCP1 expression level at the cell surface 72 h after siRNA transfection. Filled area, unstained HpSCs; blue line, untransfected HpSCs; red line, HpSCs transfected with negative control siRNA; black line, HpSCs transfected with siCDCP1. **c**, **d** Representative images of the proliferation of siRNA-treated HpSCs (**c**) and cell number counts (**d**) 96 h after cell plating onto 24-well plates at a density of 2000 cells/well. Scale bars: 100 μm. Results shown as mean cell number count ± SD (*n* = 3 independent experiments). Mann–Whitney test, ***P < 0.0001, NS no significant difference. **e**, **f** Viability of siRNA-treated HpSCs determined by colony-formation assay. Representative images of colonies are shown in (**c**). Lower lane shows a magnified image of the upper lane. Colony number counts and cell number counts in each colony are shown in (**f**). HpSCs transfected with negative control siRNA and siCDCP1 were seeded onto 24-well plates at a density of 400 cells/well, photographs taken, and colony number counts and cell number counts were performed 96 h after cell plating. Con, untransfected HpSCs; siNC, HpSCs transfected with negative control siRNA; siCDCP1, HpSCs transfected with siCDCP1. Scale bars: 100 μm. Results shown as mean ± SD (*n* = 3 independent experiments). Mann–Whitney test, *P < 0.05, **P < 0.001, ***P < 0.0001, NS no significant difference. See also Additional file 4: Figure S4. CDCP1 CUB domain-containing protein 1

To elucidate whether CDCP1 is essential for the self-renewal of HpSCs in culture, we performed loss-of-function assays. A single CDCP1^+^CD90^+^CD66^–^ HpSC-derived colony was subcultured and transfected with CDCP1-siRNA (siCDCP1), and knockdown of the *CDCP1* mRNA expression level (Fig. 5a) and CDCP1 protein level (Fig. 5b) was observed. We tested for differences in the proliferation rate between transfected cells and control cells. The siCDCP1 cells grew slowly and showed growth inhibition, with about half the cell numbers compared to cells without CDCP1 inhibition (Fig. 5c, d). The self-renewal capability of siCDCP1 cells was also examined with a colony formation assay. siCDCP1 in HpSCs resulted in an approximate 3-fold decrease in colony formation efficiency, and the generated colony size was significantly smaller than the control (Fig. 5e, f). In addition, the migratory activity of HpSCs was suppressed by siRNA-mediated downregulation of CDCP1 in HpSCs (Additional file 4: Figure S4A, B). These results indicate that CDCP1 is a key regulator of proliferation/self-renewal and migration in HpSCs. Taken together, these data demonstrate that CDCP1^+^CD90^+^CD66^–^ HpSCs have a bipotential phenotype and self-renewal capability.

### Robust repopulation of liver-injured recipients with human HpSCs

**Fig. 6:**
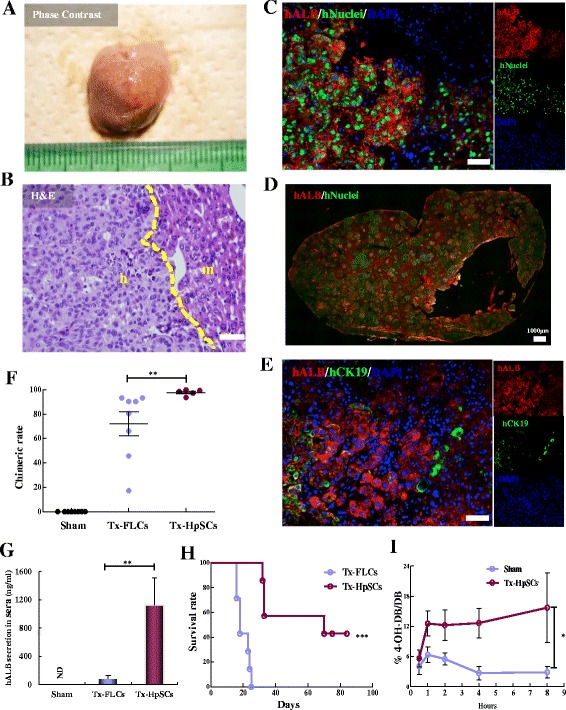
Engraftment and repopulation of uPA-NOG mouse liver with human HpSCs. **a** Macrophotography of a humanized liver 1 month after being transplanted with human HpSCs. **b** Human HpSC-derived liver structure in a humanized liver lobe (H&E). m mouse liver region, h human HpSC-derived liver region. Scale bar: 50 μm. **c**, **d** Immunohistochemistry used to distinguish human liver cells from mouse liver cells with anti-human ALB (red) and anti-human nuclear antigen (green). Nuclei stained with DAPI (blue). Scale bars: 50 μm (**c**) and 1000 μm (**d**). **e** Immunostaining of humanized liver with human ALB (red) and human CK19 (green). Nuclei stained with DAPI (blue). Scale bar: 50 μm. Results representative of at least three independent experiments. **f** Chimeric rate in uPA-NOG mice 1 month after donor cell transplantation. Data represent mean ± SEM (*n* = 8 independent experiments). Mann–Whitney test, **P = 0.0042 between human primary FLCs and human HpSCs. **g** Human albumin detection in mouse sera 1 month after donor cell transplantation. Data shown as mean ± SD (*n* = 5 independent experiments). Mann–Whitney test, *P* = 0.0079 between human primary FLCs and human HpSCs. ND undetectable. **h** Kaplan–Meier survival curve of uPA-NOG mice after human primary FLC and HpSC transplantation (*n* = 7 in each group). Log-rank (Mantel–Cox) test, *P* = 0.0001. **i** Pharmacokinetic analysis of serum metabolite 4-OH debrisoquine formation to debrisoquine in sera of human HpSC-derived humanized mice and sham mice. Data shown as mean value ± SEM (*n* = 3 independent experiments). Mann–Whitney test, *P* = 0.0159. See also Additional file 5: Figure S5. H&E hematoxylin and eosin, hALB human albumin, HpSC hepatic stem cell, FLC primary fetal liver cell, Tx treatment, CK cytokeratin, 4-OH-DB/DB 4-hydroxy-debrisoquine to debrisoquine

Karyotype analyses were performed on expanded HpSCs of passage 8 and were found to be normal (Additional file 5: Figure S5A). Then, to examine whether human HpSCs could expand and repopulate damaged liver tissue after transplantation, we transplanted 10^6^ cells into albumin promoter-driven urokinase-type plasminogen activator (uPA)-transgenic mice produced in severely immunodeficient NOD/Shi-scid IL2Rg^null^ (NOG) mice (termed uPA-NOG mice) [45]. One month after transplantation, morphological and histological analyses showed that human HpSCs were dramatically expanded in the mouse liver (Fig. 6a, b, Additional file 5: Figure S5B).

To determine whether human HpSCs matured after transplantation, we immunohistochemically assessed for human nuclei, human albumin (ALB), and human cytokeratin 19 (CK19) expression. The results showed that human HpSC-derived human hepatocytes in mouse livers were well differentiated with double positive for human nuclei and human ALB (Fig. 6c, d). Human ALB-positive hepatocytes that were CK19-negative resembled more matured hepatocytes, while cells that positively costained with human ALB and CK19 exhibited a bipotential capability with differentiation into hepatocytes and cholangiocytes (Fig. 6e). The repopulation level reached up to 90% 1 month after transplantation (Fig. 6f). All transplanted mice livers were assessed for human cells using an antibody against the human leukocyte antigens A, B, C (HLA) and mouse cells using an antibody to the mouse MHC H2Kd (H2kd) antigen that is specific for uPA-NOG mice. About 85% of the cells in the liver were positive for HLA and negative for H2Kd antigen (HLA^+^H2Kd^–^), indicating high human HpSC cell repopulation and no cell fusion in engrafted uPA-NOG mice (Additional file 5: Figure S5C). This suggests that CDCP1^+^CD90^+^CD66^–^ HpSCs could successfully reconstitute the mouse liver without cell fusion and exhibited a high potential for differentiation into more matured hepatocytes in vivo.

Furthermore, on the protein level, uPA-NOG mice transplanted with human HpSCs secreted more human albumin compared with mice transplanted with human primary FLCs (Fig. 6g). Additionally, all of the four mice transplanted with human primary FLCs died within 1 month post transplantation, while all of the seven mice transplanted with human HpSCs survived more than 1 month. Three of them (43%) were still alive at over 60 days post transplantation (Fig. 6h), suggesting that human HpSCs were superior to human primary FLCs in terms of repopulation efficiency, post-transplant maturation, and therapeutic recovery of the damaged liver. In addition, 2 months after human HpSC transplantation, the ratio of 4-hydroxy-debrisoquine to debrisoquine in human HpSC-derived humanized mouse livers was significantly increased from 1 to 8 h after treatment with debrisoquine, indicating that human HpSCs underwent a maturation process and achieved certain activity in vivo (Fig. 6i). Since human primary FLC derived-humanized mice died within 1 month post transplantation, we could not perform debrisoquine metabolic assays. All of the data demonstrated that transplanted human HpSCs could efficiently repopulate recipient mouse livers and had the potential to differentiate into functional hepatocytes with drug metabolism activity in uPA-NOG mice.

## Discussion

Because hepatocyte transplantation for the treatment of liver diseases is considered a viable alternative to organ transplantation due to the severe shortage of donor livers, researchers have been attempting to identify appropriate hepatocyte candidates for clinical and pharmaceutical use. Current conversion strategies are often unable to fully specify a defined cell fate. HpSCs have the potential to provide an unlimited source for transplantable hepatocytes. However, it is still unclear whether HpSCs exist in adult tissue [46]. In our study, we used FACS for cell surface antigen screening, microarray, single cell tracing, colony forming assays and siRNA transfection, BrdU-labeling, and serial cell sorting to confirm the stem cell characteristics of the CDCP1^+^CD90^+^CD66^–^ subpopulation in human primary FLCs. Our work revealed that human primary FLCs expressing CDCP1^+^CD90^+^CD66^–^ constituted a pool of HpSCs with self-renewal, clonal expansion, and bipotential capability in vitro as well as in-vivo engraftment that could fully proliferate and mature for months after transplantation in mouse liver.

Many surface markers have been reported to be useful for human HpSC isolation. Schmelzer et al. [16] and Tanaka et al. (2009) [47] reported that EpCAM^+^ cells have stem cell self-renewal and colony-forming capability compared with EpCAM^–^ cells in the ductal plates in fetal liver. Our HpSCs isolated from human fetuses at 14–18 weeks of gestation showed a low EpCAM frequency. These results are similar to a report by Schmelzer et al. [16], who found that only 5% of EpCAM^+^ cells in human fetuses of 16–20 weeks gestational age were HpSCs, whereas about 95% of the EpCAM^+^ cells were hepatoblasts, indicating that hepatoblasts positively express EpCAM and that EpCAM alone is not sufficient for HpSC isolation [20, 48]. Moreover, Yanai et al. (2010) [49] found that DLK was first expressed in livers from 22-week-old fetuses and was downregulated in livers from 38-week-old fetuses, indicating that DLK is not a broad HpSC marker throughout the development process. In our study, besides CD90, we report the first use of CDCP1 as a marker for human HpSC isolation. We detected that the expression of the *CDCP1* gene in human HpSC was about 37-fold higher than that in human adult livers; moreover, our previous data demonstrated that there was a difference in CDCP1 expression during different stages of development. Indeed, CDCP1 expression in human iPS-derived liver buds was over 200-fold higher than that in human iPS cells [50], implying the specificity and origin of CDCP1 in human HpSC identification. In addition, freshly sorted CDCP1^+^CD90^+^CD66 HpSCs and single CDCP1^+^CD90^+^CD66^–^ HpSC-derived clones on culture day 14 were negative for AFP expression, but could be matured into AFP-positive hepatocytes on culture day 40, indicating that the main difference between CDCP1^+^CD90^+^CD66^–^ HpSCs and hepatoblasts is expression of AFP [51]. Previous reports showed that AFP-negative HpSCs were the precursors of hepatoblasts [51], implying that CDCP1^+^CD90^+^CD66^–^ HpSCs might represent the precursors of hepatoblasts. Furthermore, sorted CDCP1^+^CD90^+^CD66^–^ HpSCs maintained stable rates of CDCP1^+^CD90^+^ cells after up to four serial sortings, indicating that their self-renewal capabilities could be maintained in vitro. Single CDCP1^+^CD90^+^CD66^–^ HpSCs could give rise to cells containing both hepatocyte and cholangiocyte characteristics verified by positive staining with HpSC markers (ALB, AFP, CK7, and CK19), and long-term culture yielded HpSCs with mature hepatocyte functions, including glycogen storage and hepatocyte metabolism-related protein induction. To our knowledge, this is the first report describing the isolation of self-renewing and bipotent human HpSCs using CDCP1.

To study whether CDCP1^+^CD90^+^CD66^–^ HpSCs may be applicable in cell-based therapies, we examined the in-vivo engraftment capacity of CDCP1^+^CD90^+^CD66^–^ HpSCs. Thirty days after transplantation, human HpSC-derived clonal clusters were detected easily in recipient livers. In comparison with transplantation of human primary FLCs, transplantation of human HpSCs yielded a higher repopulation efficiency (over 90% on average) and exhibited prolonged longevity in recipient livers. Moreover, increased levels of human ALB were detected in human HpSC-repopulated livers, indicating that human HpSCs could be fully matured into functional hepatocytes compared with transplanted human FLCs. In addition, debrisoquine hydroxylation measurement in human HpSC-derived humanized livers also confirmed that human HpSC-repopulated livers had normal drug metabolism capacity.

## Conclusions

These data provide evidence that CDCP1 acts as a specific marker of human HpSCs and that application of CDCP1 for identifying human HpSCs for isolation may contribute to regenerative therapies of liver diseases and to understanding the mechanism of organogenesis.

## Methods

### Animal model and human primary liver cells

Albumin promoter-driven uPA-transgenic mice produced in severely immunodeficient NOG mice (referred to as uPA-NOG mice) were supplied by Laboratory Animals Research Department, Central Institute for Experimental Animals (Kawasaki, Japan). Animals were maintained and operated upon in accordance with protocols approved by the Laboratory Animal Resource Center of Yokohama City University (No. FA-14-075).

Human primary FLCs were obtained from Cell Systems (Cat No. CS-ABI-3716; Kirkland, WA, USA), and were separated and pooled from five human fetus between embryonic weeks 14 and 18 by the Applied Cell Biology Research Institute (ACBRI) with donor permission.

### Cell culture and BrdU labeling

We used a low-density clonogenic culture system for human primary FLCs as described previously [17], with some modifications. Human primary FLCs and/or flow cytometry-fractionated cells were plated in DMEM nutrient mixture F-12 Ham (DMEM/F12 1:1 mixture; Sigma, St. Louis, MO, USA) supplemented with 10% fetal bovine serum (FBS), human γ-insulin (1.0 μg/ml; Wako, Japan), nicotinamide (10 mM, Sigma), dexamethasone (100 nM, Sigma), and l-glutamine (2 mM, Gibco BRL) in dishes coated with type IV collagen (Becton Dickinson Labware) for at least 2 weeks with complete medium changes every 5 days. Growth factors were added in the first 24-h culture of human recombinant hepatocyte growth factor (HGF, 50 ng/ml; Sigma) and epidermal growth factor (EGF, 10 ng/ml; Sigma).

For passaging, culture medium was removed, and cells were treated with 0.05% trypsin–EDTA (Gibco) at room temperature for 5 min and then gently detached from the dish. Suspended cells were neutralized and washed with culture medium containing 10% FBS. The viability of dissociated cells was never lower than 90% by trypan blue exclusion detection. The plating cell density of cells varied depending on the experimental design.

For 3D culture, 3D chips (gifts from Kurarey Co. Ltd, Tsukuba, Japan) were precoated with mouse collagen type IV (BD Biosciences, Bedford, MA, USA) according to the manufacturer’s instructions. Cells were cultured on 3D chips at a density of 7.5 × 10^4^ cells/cm^2^.

BrdU-pulsed cells were collected after incubation with BrdU at a final concentration of 10 μM in cell culture medium. Multicolor immunofluorescent staining of cell surface antigens was performed using a BrdU flow kit (BD Pharmingen, San Jose, CA, USA) according to the manufacturer’s instructions.

### Antigenic profiling and sorting with flow cytometry

Suspended cells were incubated with fluorochrome-conjugated monoclonal antibodies (mAbs) on ice for 30 min in the dark. PBS with 2% FBS was used as a washing solution and antibody diluant. A two-step incubation was performed with biotinylated primary antibodies and a streptavidin-labeled fluorochrome reaction. All labeled mAbs and labeled fluorochromes were purchased from Becton Dickinson unless otherwise indicated. The following mAbs and fluorochromes were used as primary antibodies: fluorescein isothiocyanate (FITC)-conjugated anti-human CD66 (hCD66FITC), hCD13FITC, hCD24FITC, hCD29FITC, and hEpCAMFITC (Miltenyi Biotec); allophycocyanin (APC)-conjugated hCD45 (hCD45APC), hCD90APC, hCD81APC, hCD117APC, hLGR5APC (Miltenyi Biotec), and hCD133/2APC (Miltenyi Biotec); phycoerythrin (PE)-conjugated hCDCP1 (hCDCP1PE), hCD34PE, hCD44PE, hCD49fPE, hCD140aPE, hCD166PE, and hCD138PE (Miltenyi Biotec); biotinylated hCD54 (hCD54Bio; eBioscience), hCD55Bio, and hCD56Bio; hDLK (IgG1, Abcam); and rabbit anti-LDLR (Abcam). Secondary antibodies were as follows: streptavidin-labeled APC, goat anti-mouse IgG1-APC, and goat anti-rabbit IgG-Alexa 488 (Invitrogen, Carlsbad, CA, USA). Cell profiling analyses and sorting were performed on a high-speed cell sorter, MoFlo (DakoCytomation).

### Statistics

The statistical significance of differences was evaluated by the Mann–Whitney *U* test when two groups were compared or by one-way analysis of variance (ANOVA) and Bonferroni’s multiple comparison test when multiple groups were compared. We used the log-rank (Mantel–Cox) test and Kaplan–Meier method to assess post-transplantation survival. *P* < 0.05 was considered statistically significant, Statistical analysis was performed using Graphpad Prism. Additional experimental procedures are listed in Additional file 6.

### Accession numbers

The microarray data utilized in this study were deposited under the GEO accession numbers GSE62933 and GSE 62998 for Additional file 3: Figure S3.

## Additional files

Additional file 1: Figure S1. Showing stem cell characteristics of human primary FLCs, related to Fig. 1. A. Representative images of human primary FLCs under low-density culture from 100–500 cells/cm2. Clonogenicity was observed at a density lower than 200 cells/cm2. The white-boxed image is a magnified image. Numbers represent the plating cell density. Scale bars: 200 μm. B. Immunofluorescence labeling of human primary FLCs with human CK19 (green, left), human AFP (red, left), human CK19 (green, right), and human ALB (red, right). The lower lane shows a magnified image of the upper lane. Nuclei were counterstained with DAPI. Scale bars: 50 μm. C. Flow cytometry was used to analyze the CD49f+/lowCD29+ hepatic stem cell fraction between mouse primary FLCs and human primary FLCs. The framed subpopulation shows the previously reported CD49f+/lowCD29+ hepatic stem cell population in mouse primary FLCs and human primary FLCs. D. Representative FACS histogram plots of human primary FLCs for stem cell-related markers. Percentages indicate positive cells that express each respective marker, with unstained control cells (filled histogram) and cells stained with antibodies against the surface proteins (empty histogram). (PDF 725 kb)

Additional file 2: Figure S2. Showing characteristics of putative CDCP1^+^CD90^+^CD66^–^ HpSCs, related to Fig. 1. Immunophenotype of HpSCs after 7 days in culture. Representative flow cytometry histograms of stem cell-related surface markers CD24, CD49f, CD44, CD55, CD166, CD54, CD117, CD138, CD140a, EpCAM, CD34, DLK, and CD13, and the hepatic C virus receptors CD81 and LDLR. Percentages indicate positive cells that express each respective marker, with unstained control cells (filled histogram) and cells stained with antibodies against the surface proteins (empty histogram). (PDF 78 kb)

Additional file 3: Figure S3. Showing microarray analysis and identification of CDCP1^+^CD90^+^CD66^–^ HpSCs, related to Fig. 1. Heatmap view of (**A**) the Wnt signaling pathway (GO:0016055) (raw signal > 1000), (**B**) plasma membrane part (GO:0044459) (more than 3-fold changes in both AH vs HpSCs and FLCs vs HpSCs), and (**C**) stemness and other related genes. HpSCs-1 and HpSCs-2 represent FACS-sorted fresh CDCP1^+^CD90^+^CD66^–^ HpSCs; FLCs represent samples from human primary FLCs; AH-1 and AH-2 represent samples from human adult liver cells. (PDF 203 kb)

Additional file 4: Figure S4. Showing CDCP1 knockdown blocks HpSC migration, related to Fig. 5. **A** Migration of HpSCs was evaluated using transwell chambers. HpSCs transfected with CDCP1 siRNA, negative control siRNA, or untreated HpSCs were plated 24 h after transfection on 24-well transwell plates. Cells that migrated through the pores to the under surface of the membrane were counted. Lower lane shows a magnified image of the upper lane. Scale bars: 100 μm. **B** Quantification of the migrated cell numbers. Con, untransfected HpSCs; siNC, HpSCs transfected with negative control siRNA; siCDCP1, HpSCs transfected with siCDCP1. Results shown as mean ± SD (*n* = 3 independent experiments). Mann–Whitney test, *P < 0.05, NS no significant difference. (PDF 292 kb)

Additional file 5: Figure S5. Showing assessment of in-vivo engrafted human HpSCs in uPA-NOG mice, related to Fig. 6. **A** Representative karyotyping image of expanded HpSC cultured for 50 days (P8), illustrating a normal chromosomal count (*n* = 46). **B** Image of a macroscopic whole mouse liver 1 month after transplantation of human BMI1-overexpressing HpSCs. GFP fluorescence shows human EGFP-HpSCs. **C** Flow cytometric analysis of mice liver engrafted with human HpSCs. Cells were analyzed with human HLA-ABC and mouse H2K^d^ expression in dissociated humanized livers of 1 month. HLA antibody does not cross-react with mouse cells (*n* = 3 independent experiments). (PDF 134 kb)

Additional file 6: Contains supplementary material and methods, including RNA interference, induction of cholangiocytic cyst formation by HpSCs, retroviral vector construction and transduction, histochemistry and immunohistochemistry, real-time PCR (qPCR), hepatic function assays, cell transplantation, microarray, flow cytometric analysis of transplanted liver cells obtained by collagenase perfusion, human ALB detection, and drug metabolite detection. (PDF 366 kb)

## Abbreviations

AAT: Alpha-1 antitrypsin
AFP: Alpha-fetoprotein
ALB: Albumin
BrdU: 5-Bromo-2-deoxyuridine
CDCP1: CUB domain-containing protein 1
CFU-C: Colony-forming unit cell
CK19: Cytokeratin 19
CK7: Cytokeratin 7
FACS: Fluorescence-activated cell sorting
FLC: Primary fetal liver cell
HLA: Human leukocyte antigens A, B, C
HpSC: Hepatic stem cell
siCDCP1 (CDCP1-siRNA): CDCP1 small interfering RNA
TAT: Tyrosine aminotransferase
