# Supplementary material for: Hepatic stem cells with self-renewal and liver repopulation potential are harbored in CDCP1-positive subpopulations of human fetal liver cells
Source: Stem Cell Res Ther. 2018 Feb 5;9:29. doi: 10.1186/s13287-017-0747-3 (PMC5800061; doi:10.1186/s13287-017-0747-3)
Supplement: Supplementary file 1 — Showing stem cell characteristics of human primary FLCs, related to Fig. 1. A. Representative images of human primary FLCs under low-density culture from 100–500 cells/cm2. Clonogenicity was observed at a density lower than 200 cells/cm2. The white-boxed image is a magnified image. Numbers represent the plating cell density. Scale bars: 200 μm. B. Immunofluorescence labeling of human primary FLCs with human CK19 (green, left), human AFP (red, left), human CK19 (green, right), and human ALB (red, right). The lower lane shows a magnified image of the upper lane. Nuclei were counterstained with DAPI. Scale bars: 50 μm. C. Flow cytometry was used to analyze the CD49f+/lowCD29+ hepatic stem cell fraction between mouse primary FLCs and human primary FLCs. The framed subpopulation shows the previously reported CD49f+/lowCD29+ hepatic stem cell population in mouse primary FLCs and human primary FLCs. D. Representative FACS histogram plots of human primary FLCs for stem cell-related markers. Percentages indicate positive cells that express each respective marker, with unstained control cells (filled histogram) and cells stained with antibodies against the surface proteins (empty histogram). (PDF 725 kb) [file 13287_2017_747_MOESM1_ESM.pdf]

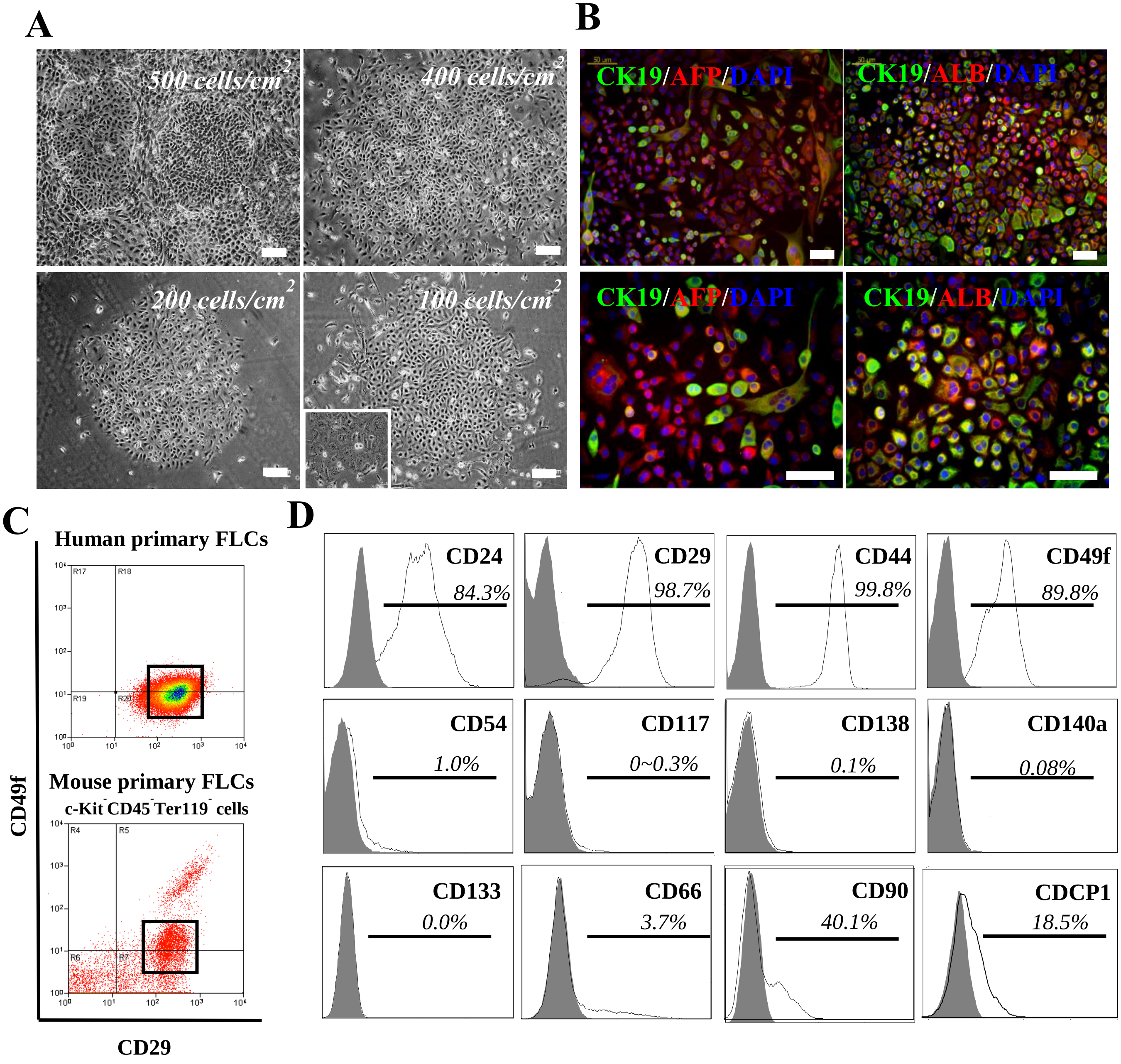


**Figure S1. Stem cell characteristics of human primary FLCs, Related to Figure 1.** A. Representative images of human primary FLCs under low-density culture from 100–500 cells/cm$^2$. Clonogenicity was observed at a density lower than 200 cells/cm$^2$. The white-boxed image is a magnified image. Numbers represent the plating cell density. Scale bars: 200 μm. B. Immunofluorescence labeling of human primary FLCs with human CK19 (green, left), human AFP (red, left), human CK19 (green, right), and human

ALB (red, right). The lower lane shows a magnified image of the upper lane. Nuclei were counterstained with DAPI. Scale bars: 50 μm. C. Flow cytometry was used to analyze the $CD49f^{+/low}CD29^{+}$ hepatic stem cell fraction between mouse primary FLCs and human primary FLCs. The framed subpopulation shows the previously reported $CD49f^{+/low}CD29^{+}$ hepatic stem cell population in mouse primary FLCs and human primary FLCs. D. Representative FACS histogram plots of human primary FLCs for stem cell-related markers. Percentages indicate positive cells that express each respective marker, with unstained control cells (filled histogram) and cells stained with antibodies against the surface proteins (empty histogram).
