# Supplementary material for: Hepatic stem cells with self-renewal and liver repopulation potential are harbored in CDCP1-positive subpopulations of human fetal liver cells
Source: Stem Cell Res Ther. 2018 Feb 5;9:29. doi: 10.1186/s13287-017-0747-3 (PMC5800061; doi:10.1186/s13287-017-0747-3)
Supplement: Supplementary file 2 — Showing characteristics of putative CDCP1+CD90+CD66– HpSCs, related to Fig. 1. Immunophenotype of HpSCs after 7 days in culture. Representative flow cytometry histograms of stem cell-related surface markers CD24, CD49f, CD44, CD55, CD166, CD54, CD117, CD138, CD140a, EpCAM, CD34, DLK, and CD13, and the hepatic C virus receptors CD81 and LDLR. Percentages indicate positive cells that express each respective marker, with unstained control cells (filled histogram) and cells stained with antibodies against the surface proteins (empty histogram). (PDF 78 kb) [file 13287_2017_747_MOESM2_ESM.pdf]

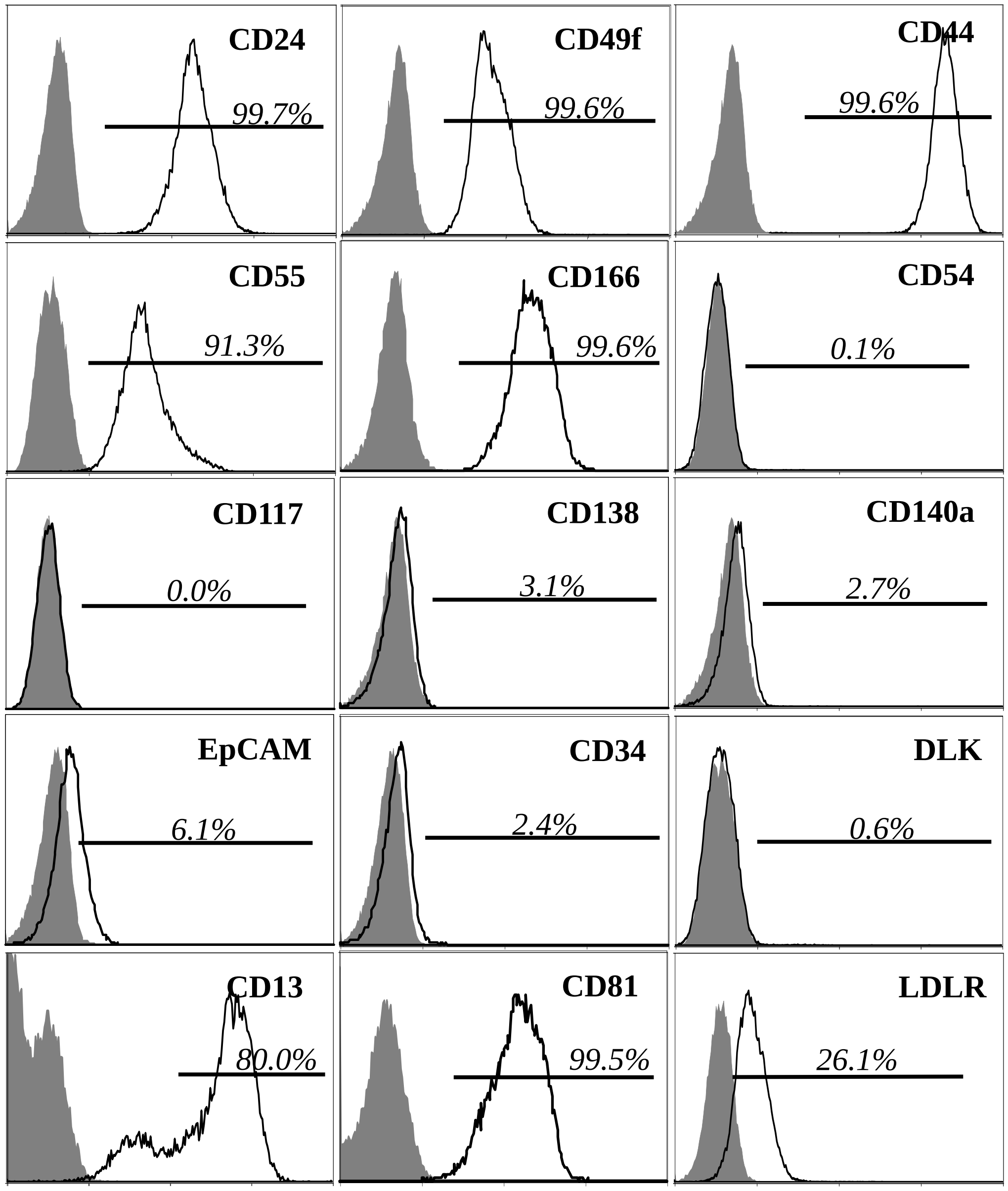

Figure S2. Characteristics of putative CDCP1$^+$CD90$^+$CD66$^-$ HpSCs, Related to Figure 1. Immunophenotype of HpSCs after 7 days in culture. Representative flow cytometry histograms of stem cell-related surface markers CD24, CD49f, CD44, CD55, CD166, CD54, CD117, CD138, CD140a, EpCAM, CD34, DLK, CD13, and the hepatic C virus receptors CD81 and LDLR are shown. Percentages indicate positive cells that express each respective marker, with unstained control cells (filled histogram) and cells stained with antibodies against the surface proteins (empty histogram).
