# Supplementary material for: Hepatic stem cells with self-renewal and liver repopulation potential are harbored in CDCP1-positive subpopulations of human fetal liver cells
Source: Stem Cell Res Ther. 2018 Feb 5;9:29. doi: 10.1186/s13287-017-0747-3 (PMC5800061; doi:10.1186/s13287-017-0747-3)
Supplement: Supplementary file 3 — Showing microarray analysis and identification of CDCP1+CD90+CD66– HpSCs, related to Fig. 1. Heatmap view of (A) the Wnt signaling pathway (GO:0016055) (raw signal > 1000), (B) plasma membrane part (GO:0044459) (more than 3-fold changes in both AH vs HpSCs and FLCs vs HpSCs), and (C) stemness and other related genes. HpSCs-1 and HpSCs-2 represent FACS-sorted fresh CDCP1+CD90+CD66– HpSCs; FLCs represent samples from human primary FLCs; AH-1 and AH-2 represent samples from human adult liver cells. (PDF 203 kb) [file 13287_2017_747_MOESM3_ESM.pdf]

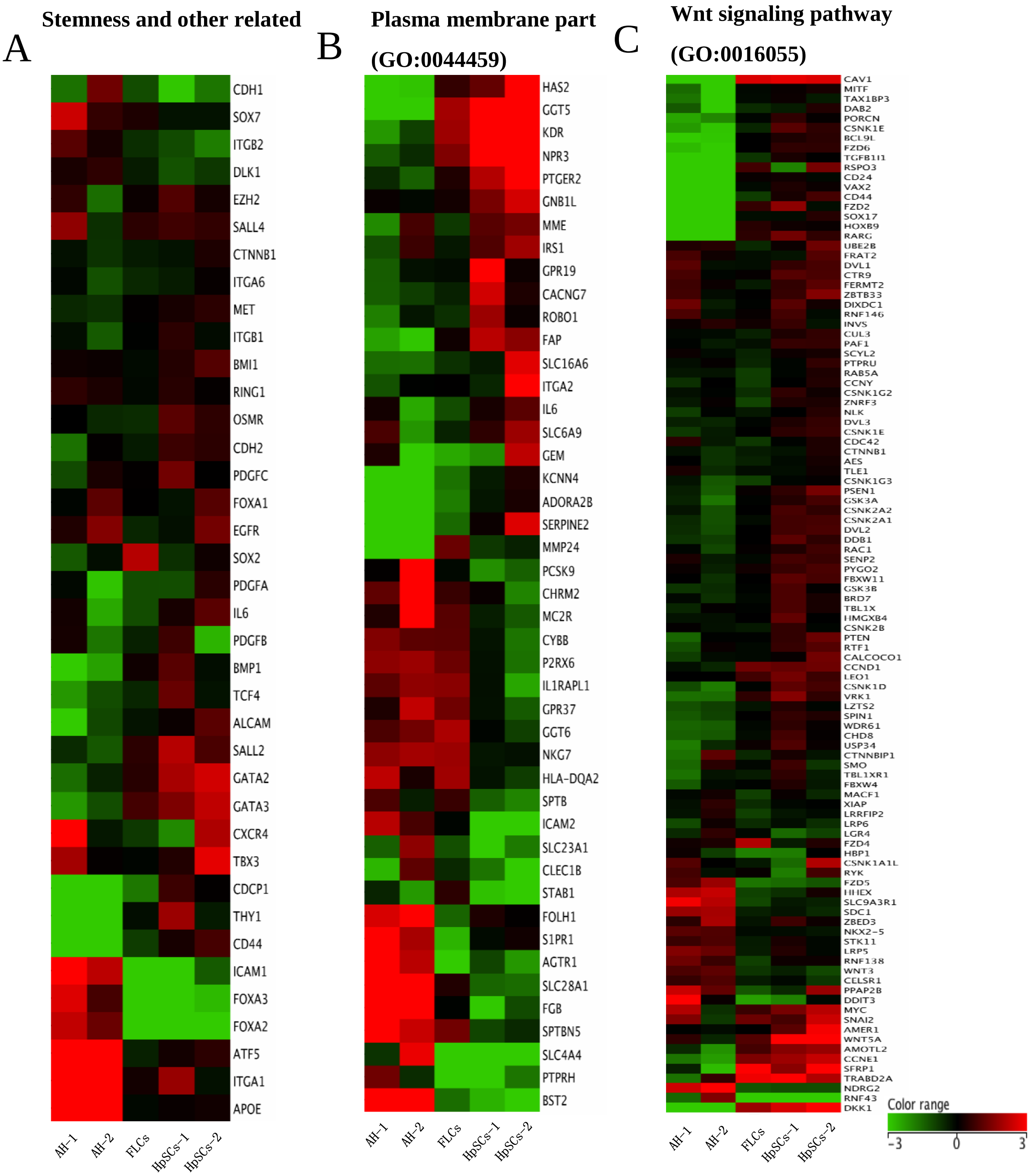

Figure S3. Microarray analysis and identification of CDCP1+CD90+CD66- HpSCs, Related to Figure 1. Heatmap view of (A) the Wnt signaling pathway (GO:0016055) (Raw signal >1000), (B) plasma membrane part (GO:0044459) (more than 3-fold changes in both AH vs. HpSCs and FLCs vs. HpSCs), and (C) stemness and other related genes. HpSCs-1 and HpSCs-2 represent FACS-sorted fresh CDCP1+CD90+CD66- HpSCs; FLCs represent samples from human primary FLCs; AH-1 and AH-2 represent samples from human adult liver cells.
