# Supplementary material for: Hepatic stem cells with self-renewal and liver repopulation potential are harbored in CDCP1-positive subpopulations of human fetal liver cells
Source: Stem Cell Res Ther. 2018 Feb 5;9:29. doi: 10.1186/s13287-017-0747-3 (PMC5800061; doi:10.1186/s13287-017-0747-3)
Supplement: Supplementary file 4 — Showing CDCP1 knockdown blocks HpSC migration, related to Fig. 5. A Migration of HpSCs was evaluated using transwell chambers. HpSCs transfected with CDCP1 siRNA, negative control siRNA, or untreated HpSCs were plated 24 h after transfection on 24-well transwell plates. Cells that migrated through the pores to the under surface of the membrane were counted. Lower lane shows a magnified image of the upper lane. Scale bars: 100 μm. B Quantification of the migrated cell numbers. Con, untransfected HpSCs; siNC, HpSCs transfected with negative control siRNA; siCDCP1, HpSCs transfected with siCDCP1. Results shown as mean ± SD (n = 3 independent experiments). Mann–Whitney test, *P < 0.05, NS no significant difference. (PDF 292 kb) [file 13287_2017_747_MOESM4_ESM.pdf]

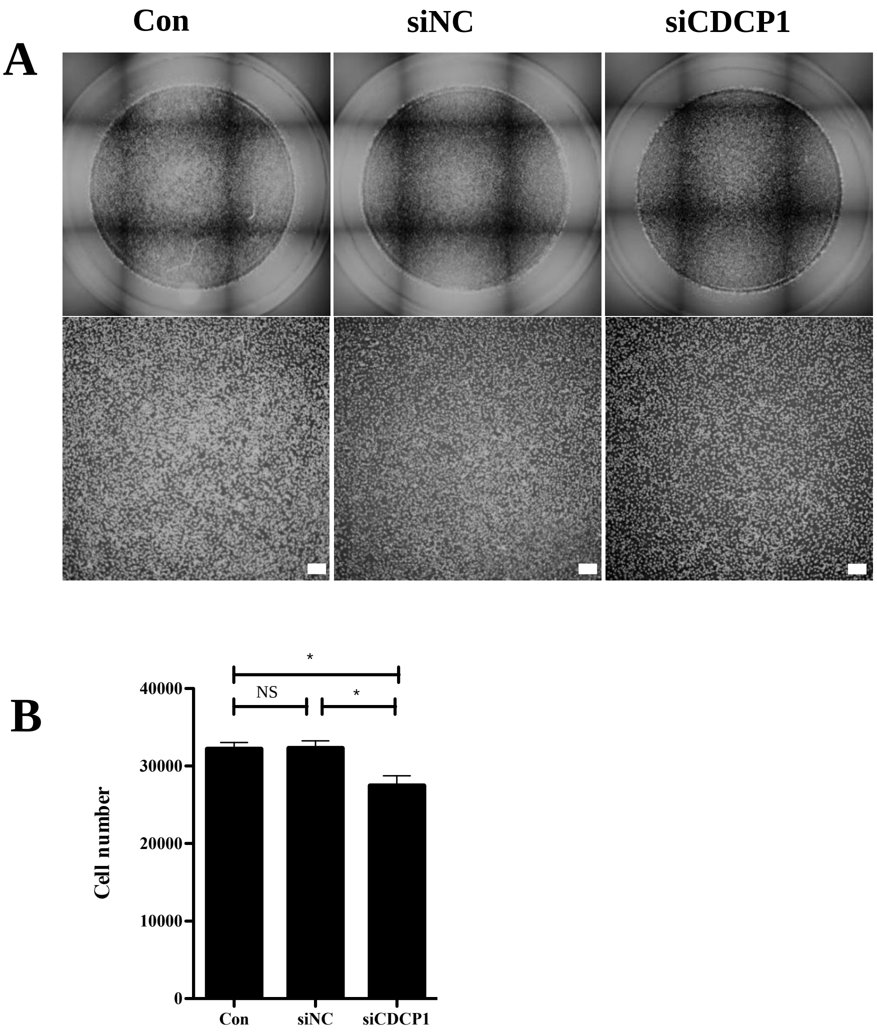

Figure S4. CDCP1 knockdown blocks HpSCs migration, Related to Figure 5. A. Migration of HpSCs was evaluated using transwell chambers. HpSCs transfected with CDCP1 siRNA, negative control siRNA, or untreated HpSCs were plated 24 h after transfection on 24-well transwell plates. Cells that migrated through the pores to the under surface of the membrane were counted. The lower lane shows a magnified image of the upper lane. Scale bars: 100 μm. B. Quantification of the migrated cell numbers. Con: untransfected HpSCs; siNC: HpSCs transfected with negative control siRNA; siCDCP1: HpSCs transfected with siCDCP1. Results are shown as the mean value ± SDs (n = 3 independent experiments). Mann-Whitney test, *$P < 0.05$, NS: no significant difference.
