# Supplementary material for: Hepatic stem cells with self-renewal and liver repopulation potential are harbored in CDCP1-positive subpopulations of human fetal liver cells
Source: Stem Cell Res Ther. 2018 Feb 5;9:29. doi: 10.1186/s13287-017-0747-3 (PMC5800061; doi:10.1186/s13287-017-0747-3)
Supplement: Supplementary file 5 — Showing assessment of in-vivo engrafted human HpSCs in uPA-NOG mice, related to Fig. 6. A Representative karyotyping image of expanded HpSC cultured for 50 days (P8), illustrating a normal chromosomal count (n = 46). B Image of a macroscopic whole mouse liver 1 month after transplantation of human BMI1-overexpressing HpSCs. GFP fluorescence shows human EGFP-HpSCs. C Flow cytometric analysis of mice liver engrafted with human HpSCs. Cells were analyzed with human HLA-ABC and mouse H2Kd expression in dissociated humanized livers of 1 month. HLA antibody does not cross-react with mouse cells (n = 3 independent experiments). (PDF 134 kb) [file 13287_2017_747_MOESM5_ESM.pdf]

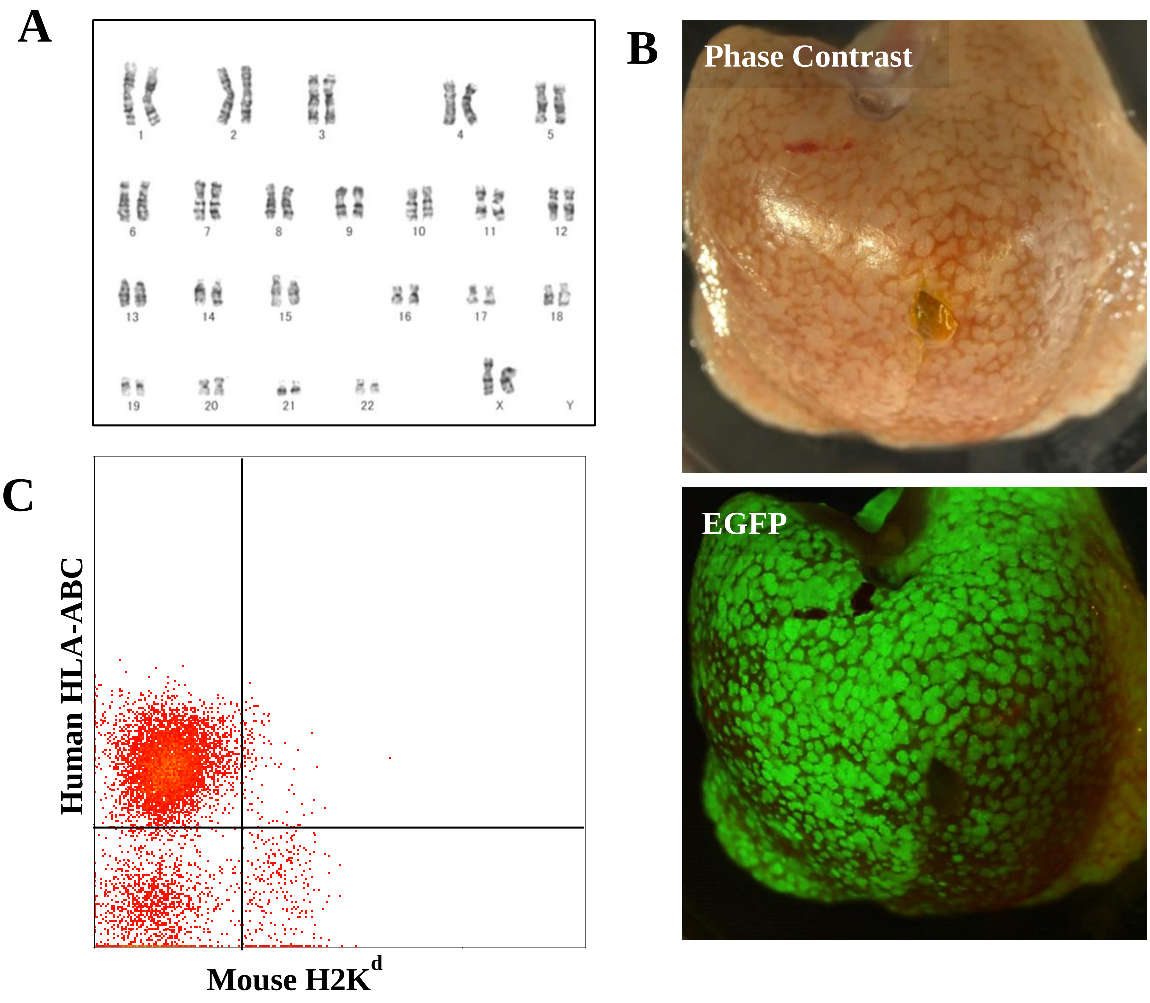

Figure S5. Assessment of in vivo engrafted human HpSCs in uPA-NOG mice, Related to Figure 6. A. Representative karyotyping image of expanded HpSC cultured for 50 days (P8), illustrating a normal chromosomal count (n = 46). B. Image of a macroscopic whole mouse liver 1 month after transplantation of human BMI1-overexpressing HpSCs. GFP fluorescence shows human EGFP-HpSCs. C. Flow cytometric analysis of mice liver engrafted with human HpSCs. Cells were analyzed with human HLA-ABC and mouse H2K^d expression in dissociated humanized livers of 1 month. The HLA antibody does not cross react with mouse cells (n = 3 independent experiments).
