# Supplementary material for: Hepatic stem cells with self-renewal and liver repopulation potential are harbored in CDCP1-positive subpopulations of human fetal liver cells
Source: Stem Cell Res Ther. 2018 Feb 5;9:29. doi: 10.1186/s13287-017-0747-3 (PMC5800061; doi:10.1186/s13287-017-0747-3)
Supplement: Supplementary file 6 — Contains supplementary material and methods, including RNA interference, induction of cholangiocytic cyst formation by HpSCs, retroviral vector construction and transduction, histochemistry and immunohistochemistry, real-time PCR (qPCR), hepatic function assays, cell transplantation, microarray, flow cytometric analysis of transplanted liver cells obtained by collagenase perfusion, human ALB detection, and drug metabolite detection. (PDF 366 kb) [file 13287_2017_747_MOESM6_ESM.pdf]

Supplementary material and methods

RNA interference

A total of 2.5×10^4 cells/well were cultured on 24-well plates. After 24 h culture, cells were transfected with CDCP1 siRNA (50 nM) or negative control siRNA (50 nM) using Lipofectamine 2000 (Invitrogen) according to the manufacturer instructions. The specific siRNA sequences for human CDCP1 and the negative control were synthesized by Sigma (Sigma-Aldrich; München, Germany). Forty-eight hours later, the total RNA of transfected cells was collected for gene analysis. For FACS analysis, the cells were collected 72 h after transfection. For cell proliferation and cell colony formation assays, cells were collected and replated 48 h after siRNA treatment. For cell proliferation, 2000 cells/well were cultured on 24-well plates, and the total cell number was counted using IN Cell Analyzer 2000 (GE Healthcare, Little Chalfont, UK) after 96 h culture. For the cell colony formation assay, 400 cells/well were seeded on 24-well plates, and the colonies were counted using IN Cell Analyzer 2000 after 96 h culture. In the transwell experiment, 24 h after cell transfection, 20,000 cells/well were seeded on an 8.0-μm transwell insert, and the migration of cells was analyzed with IN Cell Analyzer 2000 after 72 h culture. The sequences of siRNAs were as follows:

siCDCP1-1-sense: 5′-GCAAACCGCUCAUCUAUAATT-3′

siCDCP1-1-antisense: 5′-UUAUAGAUGAGCGGUUUGCTT-3′

siCDCP1-2- sense: 5′-CGGUUUAGAGCUGCAGUUUTT-3′

siCDCP1-2- antisense: 5′-AAACUGCAGCUCUAAACCGTT-3′

siCDCP1-3- sense: 5′-GAAAGAAUAGUCUUUACCUTT-3′

siCDCP1-3- antisense: 5′-AGGUAAAGACUAUUCUUUCTT-3′

siRNA-negative control-sense: 5′- AAUUCUCCGAACGUGUCACGU-3′

siRNA-negative control- antisense: 5′-ACGUGACACGUUCGGAGAAUU-3′

Induction of cholangiocytic cyst formation by HpSCs

Single CD318+CD90+CD66− HpSC-derived colonies were passaged and trypsinized using 0.05% trypsin-EDTA, washed in DMEM/F12 containing 10% FBS, and then counted. The cells were then combined with an extracellular matrix gel consisting of a mixture of 40% collagen type-I (Nitta Gelatin; Osaka, Japan) and 40% Matrigel (BD Biosciences; Bedford, MA, USA), and cultured in 24-well culture plates (2000 cells/50 μL extracellular matrix gel/well). After 10 min incubation, the culture medium was added, followed by incubation for 10–12 days with medium changes every 3 days. The culture medium was a 1:1 mixture of H-CFU-C medium and DMEM/F-12 supplemented with 2% B27 supplement, 0.25 mM A-83-01, 10 μM Y-27632, 20 ng/ml EGF, 40 ng/mL HGF, 40 ng/ml recombinant human Wnt-3a (R&D Systems, Minneapolis, USA), and 100 ng/ml recombinant human R-Spondin 1 (R&D Systems). H-CFU-C medium consisted of DMEM/F-12 supplemented with 10% FBS (Nichirei Biosciences; Tokyo, Japan), 1× Insulin-Transferrin-Selenium X

(Invitrogen), 10 mM nicotinamide (Sigma), 10^{-7} M dexamethasone (Sigma), 2.5 mM 2-[4-(2-hydroxyethyl)-1-piperazinyl] ethanesulfonic acid (HEPES) buffer solution (Invitrogen), 1× penicillin streptomycin glutamine, and 1× nonessential amino acids. Cysts in gels were stained and analyzed under a confocal microscope.

Retroviral vector construction and transduction

A retroviral vector with pGCDNsamIRES-EGFP was used to generate EGFP HpSCs. To produce recombinant retroviruses, plasmid DNA was transfected into 293gp cells with pCMV-VSV-G under culture conditions of 10% FBS, L-glutamine (2 mM, Gibco BRL), puromycin (2 μg/mL, Sigma), G418 (0.3 mg/mL, Sigma), 1× penicillin/streptomycin (Gibco) and incubated at 37°C with 10% CO_2 on poly-L-lysine coated dishes. After reaching confluence, the medium was replaced with DMEM without tetracycline. The virus in the supernatant was concentrated by centrifugation at 6000 × *g* at 4°C for 16 h, and the pellet was diluted with StemPro-34 Serum Free Medium (Invitrogen). Cells were transduced with the indicated viruses in the presence of protamine sulfate (6–10 μg/mL, Wako) and centrifuged at 400 × *g* at 32°C for 30 min. After incubation at 37°C with 5% CO_2 for 90 min, the medium was replaced with fresh culture medium. After incubation for 72 h, EGFP-overexpressing cells were enriched with flow cytometry.

Histochemistry and immunohistochemistry

Giemsa and Periodic acid Schiff (Wako) staining was performed in accordance with the

manufacturer's instructions. Liver tissues were embedded in OCT compound in cryomolds. Five-micron-thick cryostat sections were cut and mounted on glass slides. The slides were then stored at -80°C until use.

For hematoxylin and eosin (HE) staining, sections were fixed with 10% formalin, washed with PBS, and stained. For dual or triple immunohistochemical staining, cells or liver sections were fixed with cold methanol for 30 min and blocked with 10% normal goat serum (NGS) for 60 min. Samples were then incubated with primary antibodies (1:200; mouse anti-human albumin mAb [Sigma], mouse anti-human AFP mAb [Sigma], mouse anti-human CK19 mAb [Progen], mouse anti-human CK7 mAb [Dako], rabbit anti-human CYP3A4 [Chemicon], rabbit anti-human CYP2D6 [Chemicon], rabbit anti-human CYP1A2 [Chemicon], guinea pig anti-human CK8/18 [Progen], and mouse anti-human nuclei [Millipore]) at 4°C overnight, washed with PBS, and incubated with appropriate Alexa-488, -555, or -647-conjugated secondary antibodies (1:500; Invitrogen) at room temperature for 60 min. The cells were counterstained with DAPI and mounted with FA mounting fluid. Images were captured using a Zeiss AxioImager and microscope.

Real-time PCR (qPCR)

Total RNA from cells or cell colonies was extracted using Isogen reagent (Nippon Gene, Toyama, Japan). RNA was reverse transcribed into cDNA using a Super Script-III First-strand Synthesis System kit (Invitrogen). qPCR was performed with an ABI 7900 instrument in triplicate using SYBR Green

master mix (TaKaRa). Primer sequences used are listed in Table S1 and S2.

Hepatic function assays

To determine the ammonia metabolism of cultured cells, on culture day 21, NH_4Cl was added into a fresh culture supernatant at a concentration of 1 mM. After 6 h, ammonia removal activity in the supernatant was determined using an ammonia test kit (Wako-chem, Osaka, Japan) according to the manufacturer's instructions.

Cell transplantation

uPA-NOG mice (male, 8–10 weeks old) were used as recipients. When human primary FLCs or HpSCs reached 90% confluence, cells were detached and adjusted to a final cell concentration of 1×10^6 cells per 50 µL culture medium. Human HpSCs or primary FLCs (1×10^6) were transplanted into the spleens of uPA-NOG mice. Mice in the sham group were transplanted with 50 µL culture medium. Mice were killed 6-8 weeks after cell transplantation.

Microarray

Total RNA was prepared from human primary FLCs, human HpSCs of P0 and P10, and BMI1-overexpressing HpSCs of P10 using an RNeasy Mini Kit (Qiagen, Valencia, CA, USA). RNA for gene-expression profiling was hybridized on a Whole Human Genome Agilent 4 × 44K v2 Oligonucleotide Microarray (Agilent Technologies) according to the manufacturer's instructions. To perform cross-species comparisons of expression profiles, 26,153 expression data at the gene level were cross-

referenced to other species using HomoloGene IDs in the MGI curated data set of human-mouse orthology with Phenotype Annotations (http://www.informatics.jax.org). To generate the heat map, we used a hierarchical clustering method with Euclidean distance complete linkage on GeneSpring11.5.1 to analyze the gene-expression profiles.

Flow cytometric analysis of transplanted liver cells obtained by collagenase perfusion

Mice with human HpSCs were anesthetized, their abdomens were opened, and their inferior vena cava was cannulated. The hepatic vein and whole liver were then retrogradely perfused gently at 37°C with Hanks' buffered salt solution (HBSS; Gibco BRL, Grand Island, NY, USA) containing 2-[4-(2-hydroxyethyl)-1-piperazinyl] ethanesulfonic acid (HEPES) at a final concentration of 10 mM (Nakalai Tesque, Inc., Kyoto, Japan) and ethyleneglycoltetraacetic acid at a final concentration of 0.5 mmol/l. This was followed by perfusion with HBSS containing HEPES (final concentration of 10 mM) and collagenase D (final concentration of 200 μg/ml) (Roche Diagnostics GmbH, Mannheim, Germany). After perfusion, a homogeneous liver cell suspension was obtained by gentle mechanical dispersion and filtering through a 70 μm nylon mesh cell strainer. After centrifugation, the cell pellet was resuspended with red blood cell lysis buffer (Sigma-Aldrich, München, Germany), centrifuged and resuspended in PBS with 2% fetal calf serum. Then, the cells were stained with the following primary antibodies: APC-conjugated mouse anti-human HLA-ABC (BD PharMingen) and PE-conjugated rat anti-mouse H2Kd (BD PharMingen). After washing with Wash buffer, the cells were analyzed by flow

cytometry.

Human ALB detection

Blood samples (20 μL) were collected periodically from the tail vein, incubated for 5 min at room temperature, and then stored on melting ice for 20 min. Clotted blood was centrifuged for 10–15 min at 400 × *g* at 4°C, and the serum fraction was removed. Human ALB in the mouse serum samples was measured using a Human Albumin ELISA Quantitation Kit (Bethyl Laboratories) according to the manufacturer's instructions.

Drug metabolite detection

Debrisoquine (2 mg/kg) was orally administered to uPA-NOG mice in which human HpSCs were transplanted (n > 3). Sham-operated uPA-NOG mice were used as a control. Blood samples were collected at 0.5, 1, 2, 4, and 8 h after administration, and heparin-Na was added. Plasma was separated by centrifugation from blood. Internal standard (niflumic acid 1 μM) methanol solution (100 μL) was added to 5 μL of plasma and centrifuged (15,000 rpm, 4°C, 5 min). The supernatant was subjected to LC-MS/MS. An Acquity UltraPerformance LC system (Waters, Milford, MA, USA), equipped with an Aquity UPLC BEH C18 column (Waters), was used for the LC experiments. Chromatographic separation was achieved on Acquity UPLC BEH C18 (1.7 μm, 2.1 × 50 mm I.D.; Waters). The temperature of the column was maintained at 40°C. The mobile phase consisting of 10 mM ammonium acetate (solvent A) and acetonitrile (solvent B) was pumped at a flow rate of 0.8 mL/min according to

the following gradient schedule: 0% solvent B (0–0.2 min), a linear gradient from 0% to 30% solvent B (0.2–0.3 min), a linear gradient from 30% to 60% solvent B (0.3–0.85 min), 60% solvent B (0.85–1.15 min), a linear gradient from 60% to 100% solvent B (1.15–1.16 min), and 100% solvent B (1.16–1.5 min). The LC was connected to an API4000 system (AB SCIEX, Foster City, CA, USA), operated in positive electrospray ionization mode. The turbo gas was maintained at 450°C. Parental and/or fragment ions were filtered in the first quadrupole and dissociated in the collision cell using nitrogen as the collision gas. The area under the curve from time 0 until the last measurable plasma concentration (AUC0-t) was calculated using the linear trapezoidal rule. Metabolic ratios were determined by dividing AUC0-t of 4-hyroxydebrisoqune by AUC0-t of debrisoquine.

Table S1. List of Primers for qPCR Used in Figure 3

GENE	***Primer Squence-Forward***	***Primer Squence-Reverse***
Human *ALB*	5’-tgttgattgcctttgctcag-3’	5’-tggagactggcacacttgag-3’
Human *AFP*	5’-agcttggtggtggatgaaac -3’	5’-ccctcttcagcaaagcagac -3’
Human *G6P*	5’-gtcaacacattacctccagg-3’	5’-gagtagatgtgaccatcacg-3’
Human *CYP3A7*	5’-caaaagactctgagacccacaa-3’	5’-agccagcaaaaataaagataattga-3’
Human *CYP3A4*	5’-aacagcctgtgctggctatc-3’	5’-gatcacatccatgctgtagg-3’
Human *CK19*	5’-catgaaagctgccttggaaga-3’	5’-tgattctgccgctcactatcag-3’
Human *CK7*	5’-caggaccctcaatgagacg-3’	5’-ccagggagcgactgttgt-3’
Human *TAT*	5’-agccattgtggacaacatga-3’	5’-tagcttctaggggtgcctca-3’
Human *TDO2*	5’-tacagagcacttcagggag-3’	5’-cttcggtatccagtgtcg-3’
Human *AAT*	5’-acatttacccaaactgtccatt-3’	5’-gcttcagtccctttctcgtc-3’
Human *GGT*	5’-gcccagaagtgagagcagtt-3’	5’-tccagaaagcagctagaggg-3’
Human *CX43*	5’-gcccacatcaggtggactgt-3’	5’-aaggacaccaccagcatgaag-3’
Human *PROM1*	5’-gacccgtgagacaaagaagc-3’	5’-gccctcacacttgaccagtt-3’
Human *EPCAM*	5’-ccatgtgctggtgtgtgaa-3’	5’-tgtgttttagttcaatgatgatcca-3’
Human *DLK*	5’-gacggggagctctgtgatag-3’	5’-tcatagaggccatcgtcca-3’
Human *c-KIT*	5’-tgcttcacagaagaccatgc-3’	5’-gtgaccaacatggagtcgtg-3’
Human *CTNNB1*	5’-ccaggtggtggttaataagg-3’	5’-ctgaggagaacgcatgatag-3’
Human *c-MYC*	5’-aagactccagcgccttctctc-3’	5’-gttttccaactccgggatctg-3’
Human *BMI1*	5’-aatccccacctgatgtgtgt -3’	5’-catttttgaaaagccctgga-3’
Human *c-MET*	5’-catgccgacaagtgcagta-3’	5’-tcttgccatcattgtccaac-3’
Human *GAPDH*	5’-ctctgctcctcctgttcgac-3’	5’-ttgattttggagggatctcg-3’
